# Rheumatoid Arthritis: Refractory to Infliximab, a Tumor Necrosis Factor Inhibitor

**DOI:** 10.4021/jocmr630w

**Published:** 2011-09-26

**Authors:** Hana Maizuliana bt Solehan, Mohd Shahrir Mohamed Said, Sazliyana Shaharir, Sakthiswary Rajalingham

**Affiliations:** a Universiti Sains Islam Malaysia, Nilai, Malaysia; b Universiti Kebangsaan Malaysia, Bangi, Malaysia

## Abstract

Rheumatoid arthritis is one of the commonest autoimmune diseases. It is a chronic, progressive, systemic inflammatory disorder affecting the synovial joints and typically producing symmetrical arthritis. If left untreated, it leads to joint destruction and thus deformity and disability.

In the recent years, advances in molecular biology have led to a variety of new treatment approaches to rheumatoid arthritis and other systemic inflammatory diseases associated with autoimmunity. Anti tumor necrosis factor (TNF) agents are emerging in the frontline management of rheumatoid arthritis (RA) in the current era of biological treatment.

We presented a 46-year-old Chinese female with a history of seropositive RA for the past 22 years refractory and intolerant to multiple medications including sulphasalazine (SSZ), leflunomide, hydroxychloroquine (HCQ) and methotrexate (MTX), thus infliximab, a tumor necrosis factor (TNF) inhibitor was initiated. However, despite receiving 6 cycles of infliximab therapy, she still complained of persistent disabled multiple joint pain and swelling. This report will discuss about rheumatoid arthritis, which is refractory to infliximab (a TNF inhibitor) and its alternative.

**Keywords:**

Rheumatoid arthritis; Biologics treatment; Tumor-necrosis factor inhibitor; Infliximab

## Introduction

Rheumatoid arthritis (RA) is a chronic, progressive, destructive and disabling disease that primarily affects the peripheral joints. The joint involvement is symmetrical, may be remitting, but if uncontrolled may lead to destruction of joints due to erosion of cartilage and bone which leads to deformity. Approximately 15% of people with RA have a particularly severe form of the disease that manifests itself as unremitting pain and swelling, causing severe disability and loss of function [1].

Drug therapy for RA has developed and evolved from empirical relief of symptoms with non-steroidal anti-inflammatory drugs (NSAIDS) to targeted intervention in the immunoinflammatory process with tumor necrosis factor inhibitors. Biologic DMARDs, produced by recombinant DNA technology, generally target cytokines or their receptors or are directed against other cell surface molecules. These include anticytokine therapies, such as the TNF inhibitors: etanercept, infliximab and adalimumab, the interleukin-1 receptor antagonist, anakinra, and other biologic response modifiers such as abatacept (CTLA4-Ig) and anti-CD20 B-cell depleting monoclonal antibody, rituximab [2,3].

## Case Report

This is a case of 46-year old Chinese lady who has been suffering from seropositive rheumatoid arthritis since 1987. During the earlier years, she was treated with daily oral chloroquine and indomethacin, an NSAIDS. However she was lost to follow-up after about a year. She presented again to our rheumatology clinic in 1992 with polyarthritis and early morning stiffness. The patient had received multiple DMARDs therapies including oral methotrexate (MTX), sulphasalazine (SSZ), hydroxychloroquine (HCQ), leflunomide, and intermittent short course of oral prednisolone. However the medication was inadequate to control the symptoms as she still experienced occasional disturbing joints pain and she was unable to tolerate the gastrointestinal discomfort from high dose of SSZ. She also complained of mild itchiness on taking leflunomide and hydroxychloroquine. The dose of these medications was adjusted throughout the follow-up.

In December 2006, the patient was on oral leflunomide 20 mg/day, oral SSZ 1 g thrice a day and oral HCQ 200 mg/day when she complained of persistent multiple joints pain and swelling for more than six months. She then was planned for a tumor necrosis factor-? (TNF-??? infliximab therapy; however it was delayed until her first infliximab infusion in March 2009 due to financial settlement from welfare support.

During this waiting period, she was bridged with low dose of oral prednisolone (5 mg/day ) and oral MTX (7.5 mg weekly) was reintroduced in end of 2008. MTX was stopped initially due to suspected lung fibrosis, however high resolution computed tomography (HRCT) scan of thorax was done in May 2007 showed no evidence of lung fibrosis. Her oral MTX was optimized to 10 mg weekly during her first infliximab therapy. She received 155mg (3mg per kg) of intravenous infliximab infusion without any adverse reaction. Prior to this treatment, her visual analogue score (VAS) for her joint pain was 10/10.

She was planned for further six weekly infusions of total six cycles, however her fifth cycle of infusion was deferred as she had flare of polyarthritis with chest infection. The chest x-ray showed pneumonic changes over left lower zone. The patient was treated with intravenous antibiotic ceftriaxone and oral MTX was withheld. Oral prednisolone was increased to 15 mg/daily upon discharge. Her fifth cycle of infliximab infusion was given two weeks later. However, during the administration, she still complained of disturbing left elbow and right knee pain and swelling.

During her admission for 6th infliximab infusion in November 2009, the patient claimed her left elbow and right knee were still painful and swollen, restricting her daily activity. Her pain was slightly relieved by daily analgesic (meloxicam 7.5 mg). She gave VAS of 8/10 for the pain. However, she denied early morning stiffness or numbness and assured that she has been compliant to her oral DMARDs. She denied of any respiratory symptoms.

On examination, she was alert and pink, her blood pressure was 120/70 mmHg, pulse rate 86 beats per minute and afebrile. Her cardiovascular and abdominal examinations were normal, but lungs examination revealed fine crackles at left lower zone. Musculoskeletal assessment showed warm and tenderness of the first and second left metocarpophalangeal joints and mildly swollen second and third right metocarpophalangeal joints. Her left wrist, left elbow and knee joints were swollen, warm and tender. Her disease activity score (DAS28) was 5.

Laboratory evaluation revealed a positive rheumatoid factor, erythrocyte sediment rate and C-reactive protein were 62 mm/hour and 2.56 mg/l respectively. Total white count was 6.6 x10^9^/L , hemoglobin 11.2 g/dL and platelet 295 x 10^9^/L. Liver function test and renal profile were normal. Chest X-rays showed minimal right apical pleural thickening. MTX was not restarted in view of possibility of pulmonary fibrosis. Repeat HRCT of thorax and lung function test were arranged.

In view of insufficient response to infliximab, this patient was planned for adalimumab therapy, another TNF-inhibitor. During subsequent follow-up in rheumatology clinic, this patient had swollen hands and knees with DAS 28 score of 5.06. Currently this patient is waiting for financial approval from welfare support for adalimumab treatment.

## Discussion

Researches have led to a better understanding of the biology and pathogenesis of the disease. The pathogenesis of RA starts with T cell activation, which subsequently leads to multiple effects including activation and proliferation of synovial lining and endothelial cells, recruitment and activation of additional pro-inflammatory cells from the bone marrow and circulation, secretion of cytokines and proteases by macrophages and fibroblast-like synovial cells, and autoantibody production [1].

The aims of management for patients with RA are to prevent early damage of the joints, improve symptoms and restore the patient to normal non-RA status. The prolonged complete control of the abnormal inflammatory process is the fundamental goal [2,3]. This is important as joint damage occurs early in the course of rheumatoid arthritis; 30 percent of patients have radiographic evidence of bony erosions at the time of diagnosis, and this proportion increases to 60 percent by two years [4].

This patient was treated with infliximab for six cycles. Infliximab is a chimeric mouse or human anti-TNF-? monoclonal antibody, the first agent generated to selectively target TNF-?, which has been shown to be effective for RA. Lipsky et al 2000 had done a double-blind, placebo-controlled, randomized trial of 428 patients in Anti-Tumor necrosis factor Trial in Rheumatoid Arthritis with Concomitant Therapy (ATTRACT). The patients with active RA despite MTX therapy received placebo with MTX (MTX-only) or infliximab 3 mg/kg or 10 mg/kg every four or eight weeks with MTX (infliximab plus MTX) for 102 weeks. The result showed that infliximab plus MTX augments clinical and radiographic benefit in patients with active RA despite receiving treatment with MTX [5,7].

However, multiple studies have documented that patients seldom continue therapy for more than five years with current DMARDs because of loss of efficacy or intolerable side effects [8,9]. This patient had tried multiple anti-rheumatic medications, however their usefulness was limited due to side effects or lack of efficacy. She was still in active arthritis despite combination of DMARDs.

In the recent years, TNF is found to be a principle cytokine in the pathogenesis of RA. This has contributed in the development of agents that block these cytokines or their effects. TNF inhibitors are one of the most important advances in the history of the treatment of this disorder that include etanercept, infliximab, and adalimumab [10].

Infliximab when combined with MTX inhibits the progression of structural damage in patients with early RA during the two year period of treatment in the trial. Early intervention with infliximab in patients with active RA despite MTX therapy may provide long term benefits by preventing radiographic progression and preserving joint integrity. Infliximab is given by intravenous infusion at a dose of 3 mg/kg body weight at 0, 2 and 6 weeks and at 8-weekly intervals thereafter [11,12].

In this case, the MTX was discontinued while on infliximab treatment due to pulmonary toxicity. Clinical trials have confirmed both the efficacy and tolerability of the agent when used in patients with DMARD refractory RA, both alone and in combination with MTX [11,12]. Sixty percent of patients receiving infliximab, with or without MTX, experienced at least 20% improvement in disease activity. Importantly, co-administration of low dose MTX significantly prolonged the duration of response seen with low dose (1 mg/kg) infliximab. Co-administration of MTX with higher doses of infliximab (3 and 10 mg/kg) also prolonged response duration, although not statistically significantly [11].

However, despite treated with infliximab, the patient is still having persistent active disease. Although the therapeutic effects of anti-TNF agents are superior to conventional DMARDs, there are still nonresponders. Treatment should be withdrawn if response is not adequate within 6 months (as defined by an improvement in DAS28 score of more than 1.2 points) or if response is not maintained [13].

Laken et al have first reported the formation of infliximab–anti-infliximab complexes during infliximab infusion in non-responders with rheumatoid arthritis. The non-responders had high human anti-chimeric antibodies (HACAs) level measured by using radio-labelled infliximab, but low or not detectable in responders. High levels of HACAs are also most probably responsible for infusion reactions to infliximab [14]. Beart et al also found a shorter duration of response to infliximab with the presence of anti-infliximab level in patients with Crohn’s disease [15].

The development of HACA is detected in 40% of patients when infliximab is used alone, but is significantly reduced by an uninterrupted course of infliximab at doses of no less than 3 mg/kg and by co-administration of methotrexate [15]. Data have linked HACA to the development of infusion reactions, but it is less clear that HACA are responsible for the blunting of clinical responses to infliximab that occur with regular administration over time [15]. The immunogenicity of infliximab was reduced by the combination infliximab with high-dose MTX and it is effective and safe in long-term treatment up to 54 weeks. Infliximab in combination with MTX may be an optimal therapeutic regimen for patients with severe RA [15].

Navarro et al showed that lack of response to a first TNF antagonist does not predict the response to a second one, yet the efficacy of a second TNF antagonist is inferior to that of the first [16]. Finckh et al did an observational study that suggested patients with RA who have stopped a previous TNF inhibitor treatment because of ineffectiveness, changing to rituximab is more effective than switching to an alternative TNF inhibitor [17]. As for the latest National Institute and Health and clinical excellence (NICE) guideline stated that for secondary non-responders, a second TNF should be allowed, but the primary non-responders would be better at that stage to try a biologic with a different mechanism of action [18,19].

This patient is considered as a secondary non-responder as she had some improvement of arthritis in the earlier course of the infliximab treatment. Next plan in this patient is adalimumab therapy. Adalimumab, another TNF-inhibitor, is a recombinant human IgG1 monoclonal antibody that binds to human TNF with high affinity, both impairing cytokine binding to its receptors and lysing cells that express TNFon their surface. It is administered 40 mg SC every 2 weeks [18-21].

Rituximab is a chimeric B cell depleting monoclonal anti-CD20 antibody. It exerts its effect by binding to CD20 on B cells and causing cell lysis by both complement -dependent and antibody-dependent cell mediated cytotoxicity. In the Randomized Evaluation of Long-term efficacy of rituximab (REFLEX) study showed that patients who did not respond to previous TNF-inhibitor therapy do respond to rituximab [22].

Another biologic agent of different class is abatacept, which belongs to a new class of selective co-stimulation modulators. It is a recombinant fusion protein comprising the extracellular domain of human CTLA4 and a fragment of the Fc domain of human IgG1, which has been modified to prevent complement fixation. Abatacept, like CTLA4, competes with CD28 for CD80 and CD86 binding and thereby can be used to selectively modulate T-cell activation [23].

Other biologic approach in treating RA is by interleukin-1 (IL-1) and IL-6 inhibition. Anakinra, is a recombinant human interleukin-1 receptor antagonist and the latest experience to date is tocilizumab, a humanized monoclonal antibody specific for the IL-6 receptor (IL-6R). Currently numerous and large controlled trials, tocilizumab has been shown to have significant efficacy in patients with RA. The first-in-class IL-6R inhibiting monoclonal antibody is the ninth biologic agent approved for the treatment of RA and may be used alone or in combination with methotrexate or other DMARDs [24]. The drug is recently available in the United States since January 2010.

There are other new biologic therapies for RA that currently have progressed to clinical trials. The evolution of knowledge and understanding in immune derangements occurring in RA patients is also leading to novel approaches to modify the disease, as reflected in the numerous studies and subsequently the new development of the targeted therapies for RA.

Even though biologics are highly effective and recommended as part of treatment in RA, they are still under practiced in Malaysia due to their high cost. Physicians probably are more willing to use biologics if they are listed in the hospital formulary and if they are affordable to the patients, but the current price range is seen to be out of reach for most our patients.
